# Genome-Wide Association Analysis of Soybean Regeneration-Related Traits and Functional Exploration of Candidate Genes

**DOI:** 10.3390/plants15010110

**Published:** 2025-12-31

**Authors:** Huiyan Zhao, Xin Jin, Yide Zhang, Qi Zhang, Lina Zheng, Yang Yue, Xue Zhao, Yingpeng Han, Weili Teng

**Affiliations:** 1 Key Laboratory of Soybean Biology in Chinese Ministry of Education (Key Laboratory of Soybean Biology and Breeding/Genetics of Chinese Agriculture Ministry), Northeast Agricultural University, Harbin 150030, China; b220301020@neau.edu.cn (H.Z.); s230301902@neau.edu.cn (X.J.); s230301046@neau.edu.cn (Y.Z.); s230302096@neau.edu.cn (Y.Y.); xuezhao@neau.edu.cn (X.Z.); 2 College of Biological Engineering, Daqing Normal University, Daqing 163000, China; neauqiz@163.com; 3 Forestry Department, Fujian Forestry Vocational & Technical College, Nanping 353000, China; zlnykl@163.com

**Keywords:** soybean, genome-wide association analysis, cotyledonary node, regeneration genes

## Abstract

Using the cotyledonary node method, four traits related to callus induction rate were identified in 185 soybean germplasm resources. Cultivation of callus tissue is crucial for soybean (*Glycine max* (L.) Merr.) genetic transformation and functional genomics studies. Identifying genes associated with the induction rate of soybean callus tissue is therefore essential for biotechnological breeding and for understanding the molecular genetic mechanisms of soybean regeneration. The efficiency of genetic transformation impacts the breeding rate of soybeans, with its success rate dependent on the soybean regeneration system. Subsequently, whole genome association analysis (GWAS) and multidimensional functional validation were conducted. GWAS identified 66 significantly associated SNP loci corresponding to the four traits. Expression analysis in extreme phenotypes highlighted four candidate genes: *Glyma.12G164100* (*GmARF1*), *Glyma.12G164700* (*GmPPR*), *Glyma.02G006200* (*GmERF1*), and *Glyma.19G128800* (*GmAECC1*), which positively regulate callus formation. Overexpression and gene-editing assays in hairy roots confirmed that these genes significantly enhanced callus formation rate and density, with *GmARF1* exerting the most prominent effect. Hormone profiling revealed elevated levels of gibberellin (GA), auxin (IAA), cytokinin (CTK), and other phytohormones in transgenic lines, consistent with enhanced responsiveness to exogenous GA. Overall, the results suggest that these four candidate genes may promote soybean regeneration, with *GmARF1* showing the most pronounced effect. These results provide valuable genetic resources for improving soybean regeneration efficiency and accelerating genetic transformation-based breeding.

## 1. Introduction

Soybean genetic transformation is globally significant, with success heavily relying on effective regeneration systems. However, the crop’s limited regenerative capacity bottlenecks its biotechnological advancement. Previous studies on soybean regeneration have focused on genetic composition, hormones, culture conditions, and explant types, but research on its molecular basis, especially the governing genes and mechanisms, remains scarce [1,2,3]. Investigating regeneration-associated gene expression offers key insights to address this challenge. The genetic transformation of soybean has remained a globally significant topic, with successful transformation heavily reliant on the effective integration of receptor systems and transformation methods. This study is the first soybean study integrating GWAS and functional validation to elucidate genetic regulators of regeneration. Several soybean regeneration-related genes have been cloned and characterized. *GmESR1* overexpression accelerated seed germination and promoted shoot/root elongation in both Arabidopsis and soybean, laying the groundwork for efficient soybean transformation [4]. *GmRAV1* (an AP2/ERF transcription factor), induced by cytokinins, acts as a key positive regulator of root and stem regeneration [5]. *GmLEC1* overexpression shortened Arabidopsis plant length, while gene-edited lines showed normal growth [6]; this embryo-related gene also accelerates early development and enhances soybean regeneration by regulating embryogenesis and cell differentiation. Plants’ remarkable regenerative capacity is strongly modulated by exogenous hormone signaling [7,8,9], with hormone type and concentration key regulators of soybean explant regeneration efficiency. Eight major endogenous plant hormones have been identified [10], and specific combinations (e.g., 1 mg/L GA + 0.1 mg/L IAA) enhance soybean transformation efficiency [1]. Embryo emergence is highest at 5 mg/L exogenous ABA, whereas concentrations above 10 mg/L inhibit somatic embryo formation [11]. The method of inducing regeneration through tissue culture relies on the interaction of plant hormones, particularly auxin and cytokinin, which are primarily used for regenerating explants [12]. The molecular links between hormone signaling and key regeneration-related genes remain poorly defined. Existing studies have focused on hormone application effects rather than the underlying genetic mechanisms, creating a critical research gap. To address this, our study employs a novel strategy integrating genome-wide association analysis (GWAS) and functional validation to systematically identify genes that mediate hormone-regulated soybean regeneration, filling the gap between phenotypic observations of hormone effects and their genetic basis.

Specific transcription factors can integrate signals that lead to cell reprogramming and the reacquisition of embryonic or meristematic fate [13,14,15,16,17,18]. These transcription factors, as key developmental regulators, coordinate organized cellular spatial distribution during organ and embryonic development. In numerous plant species, their encoding genes enhance regeneration efficiency [19,20,21]. The auxin response factor (ARF) is composed of three parts: the DNA binding domain (DBD), the middle region (MR), and the C-terminal domain (CTD). The DBD is a B3-like domain that affects auxin response and directly regulates gene expression [22]. The amino acid sequence of the MR determines whether the ARF will activate or inhibit target gene expression [23]. The CTD of ARFs mediates homo-dimerization (with other ARFs) or hetero-dimerization (with Aux/IAA proteins)—a key auxin-regulated step. Pentatricopeptide repeat (PPR) proteins comprise an N-terminal signal sequence (often with mitochondrial/chloroplast localization signals regulating photosynthesis), intermediate tandem repeats (2–27 conserved PPR domains, the basis for classifying PPR families into P/PLS subgroups), and a C-terminal domain (also involved in classification). Auxin efflux carriers, including the AUX/LAX family and NRT proteins, are critical for plant growth and development by mediating auxin transport [24]. Efflux carriers include ATP-binding cassette (ABCB/PGP) transporters, WAT, and PIN-FORMED (PIN) proteins, among others [25]. PIN proteins are widely present in plants and are primarily responsible for the polar transport of auxin between adjacent cells [26].

Based on the above literature, we hypothesize that genetic variations in hormone signaling-related genes and uncharacterized quantitative trait nucleotides (QTNs) jointly regulate soybean regeneration traits. We further propose that these genes exhibit differential expression between high- and low-regeneration germplasms, and their function is modulated by exogenous hormones. The genetic basis of soybean regeneration has not been investigated with a large-sample GWAS approach so far, and functional validation of the candidate genes is also missing from the literature. In this study, we used 21,350 SNPs and 185 soybean germplasms to perform GWAS (MLM model) on four adventitious bud regeneration traits: induction, elongation, rooting, and seedling rates. qRT-PCR analysis of cotyledon nodes and clustered buds from high-/low-regeneration soybean varieties identified regeneration related candidate genes and novel QTNs associated with soybean regeneration. We performed subcellular localization and bioinformatic analyses, and evaluated candidate gene expression under various gibberellin treatments. Overexpression and knockout constructs were also generated for functional exploration.

## 2. Results

### 2.1. Phenotypic Data Analysis

A variance analysis was conducted on the statistical data of four regeneration-related evaluation indicators of soybean natural populations (Table 1). At the 0.05 level, the *p*-values for induction rate, elongation rate, rooting rate, and seedling rate were all less than 0.01, indicating that all four indicators reached a highly significant level for soybean regeneration evaluation. This suggests significant differences in the impact of these indicators on soybean regeneration in natural populations, indicating significant differences in regeneration rates among different soybean varieties. Eleven varieties with high regeneration rates were selected as potential recipients for genetic transformation (Supplementary Table S1).

Correlation analysis was conducted on four soybean regeneration evaluation indicators (Table 2), and the results showed that induction rate was significantly positively correlated with elongation rate, rooting rate was significantly positively correlated with seedling rate, elongation rate was significantly positively correlated with rooting rate and seedling rate, and rooting rate was significantly positively correlated with seedling rate. The broad sense heritability (H^2^) of the four regeneration traits ranged from 0.58 to 0.83. Among them, the seedling formation rate had the highest heritability H^2^ = 0.83, followed by the rooting rate H^2^ = 0.75, while the callus induction rate had a relatively lower heritability H^2^ = 0.58. These results indicate that soybean regeneration traits are dominated by genetic factors, and the degree of genetic control varies across different developmental stages. The high heritability characteristics provide a reliable genetic basis for the effective localization of associated loci via GWAS.

### 2.2. Phylogenetic Analysis, Genetic Structure Analysis, Principal Component Analysis, and Linkage Disequilibrium Analysis

**Figure 1:**
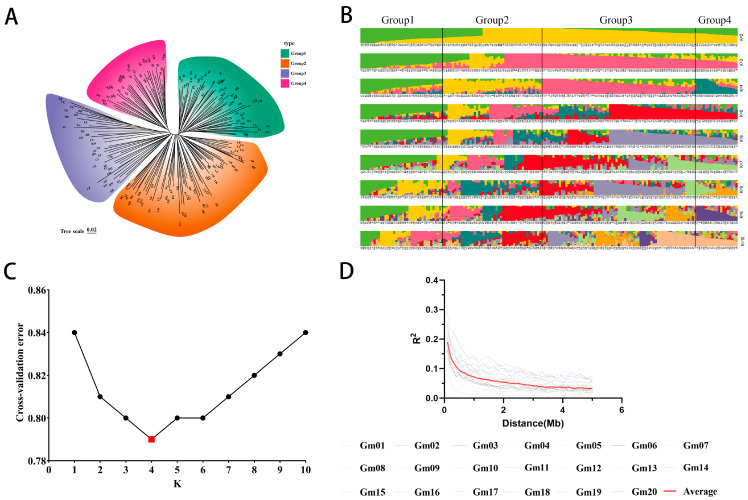
Population structure analysis for the 185 soybean accessions. (**A**) Phylogenetic tree of natural population. (**B**) Population structure analysis from k = 2 to 10. (**C**) Cross-validation error for the assumed number of populations (K) in ADMIXTURE analysis. (**D**) The genome-wide average LD decay (R^2^) of the GWAS panel.

To understand the genetic structure of 185 soybean germplasm resources, phylogenetic analysis, population structure analysis, and principal component analysis were conducted. Population structure analysis revealed that at K = 4, the 185 soybean germplasm resources were assigned to four groups (Figure 1B,C): Group 1, Group 2, Group 3, and Group 4. Group 1 consisted of 48 accessions from China, the United States, and Canada. Group 2 included 65 accessions from China, the United States, and Romania. Group 3 comprised 35 materials from China, the United States, Germany, Japan, Russia, and Ukraine. Group 4 consisted of 37 materials from China, the United States, and Italy. The neighbor-joining (NJ) tree results also suggested that the 185 soybean accessions could be divided into four groups (Figure 1A). Linkage disequilibrium decayed with increasing physical distance across all chromosomes (Figure 1D). The NJ tree analysis validated the results of the population structure analysis.

### 2.3. Distribution of SNPs Loci and Analysis of Mapping Population

**Figure 2:**
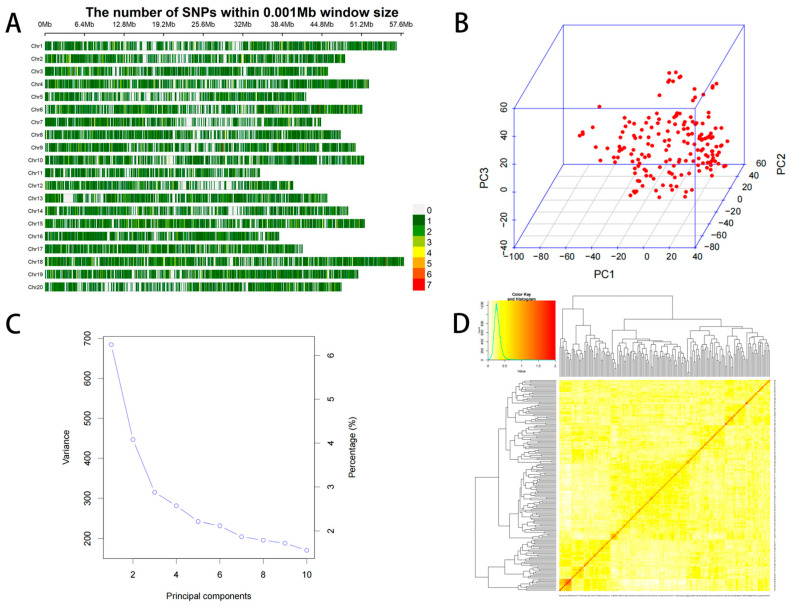
Distribution of SNPs among 20 chromosomes. (**A**) The number of SNPs within 0.001 Mb window size. (**B**) Principal component analysis (PCA) plots. (**C**) PCA (Scree plot) plot depicting the population structure of the 185 soybean genotypes. (**D**) The heat map of the kinship matrix pf 185 soybean genotypes of the current GWAS.

With a minor allele frequency (MAF) ≥ 0.05 and missing data less than 1%, a total of 23,150 single nucleotide polymorphisms (SNPs) were identified across all 20 soybean chromosomes (Figure 2A). Principal component analysis shows that there is no obvious stratification in the population, with most accessions clustering closely and a few appearing as outliers. This indicates balanced genetic relationships and genetic similarity among varieties (Figure 2B). The PCA plot of the soybean genotype population structure indicated that the first three principal components dominated the population structure (Figure 2C). The heatmap of the kinship matrix indicated that natural populations exhibited low genetic correlation (Figure 2D).

### 2.4. GWAS Analysis for Four Soybean Regeneration Traits

**Figure 3:**
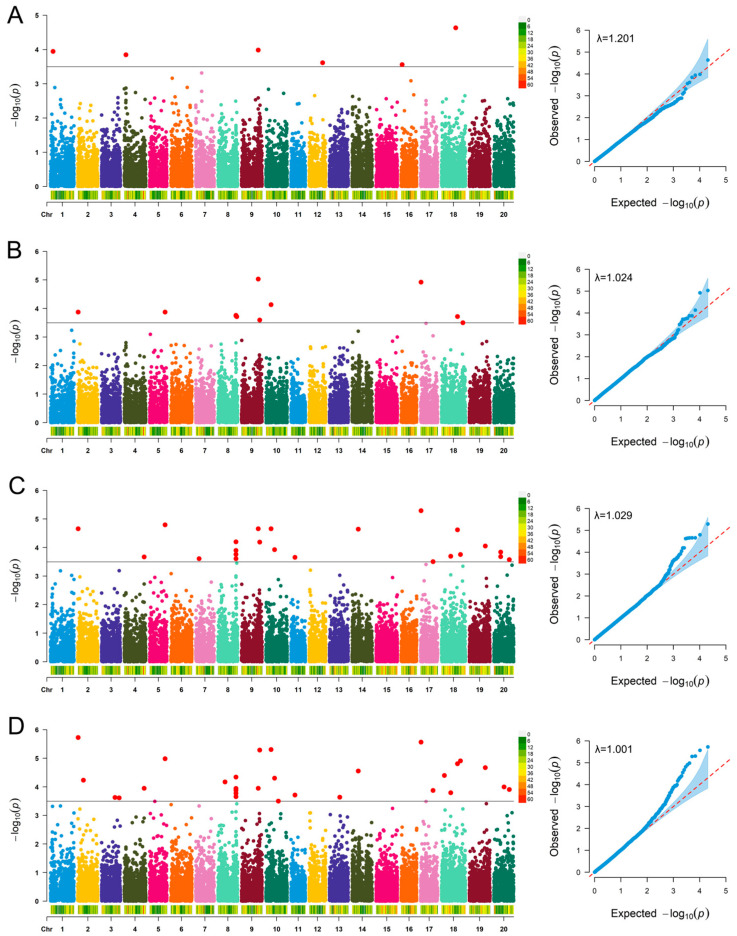
Manhattan plot of GWAS for four regeneration traits in the natural population, in which the x-axis represents the SNP along each chromosome; the y-axis is the −log_10_(*p*-value) of the association, and the threshold was set at −log (p) > 3.5 (red). (**A**): The correlation between the induction rate of clustered buds and SNP loci, (**B**): the correlation between the elongation rate of clustered buds and SNP loci, (**C**): the correlation between rooting rate and SNP loci, (**D**): the correlation between seedling rate and SNP loci.

GWAS analysis identified 66 SNPs on 12 of the 20 chromosomes that were significantly associated with regeneration at −log_10_(p) = 3.5. The threshold selected in this study is a “Suggestive association threshold”, which is designed to efficiently screen potential associated candidate regions for soybean regeneration-related traits. This avoids missing loci with weak effects but biological significance due to an excessively strict threshold, which is consistent with the genetic characteristics of soybean regeneration traits regulated by the synergistic action of multiple genes. All 66 SNP association loci (including 18 independent loci and 19 reproducible loci) screened through this suggestive association threshold are only regarded as candidate regions, and their authenticity and functional validity have been further confirmed by subsequent systematic functional verification experiments (Figure 3, Supplementary Table S2). The phenotypic variation explained by these SNPs ranged from 0.14% to 14.06%. Nineteen SNPs controlled multiple traits, including 10 SNPs that simultaneously controlled Rr and Sr, 8 SNPs that simultaneously controlled Rr, El, and Sr, and 1 SNP that simultaneously controlled Ir, Rr, El, and Sr (Table 3). Notably, the SNP rs40835778 located on chromosome 9 controlled four traits simultaneously, making it a key SNP for subsequent screening of candidate genes.

### 2.5. GO Annotation Analysis and KEGG Enrichment Analysis of Candidate Genes

**Figure 4:**
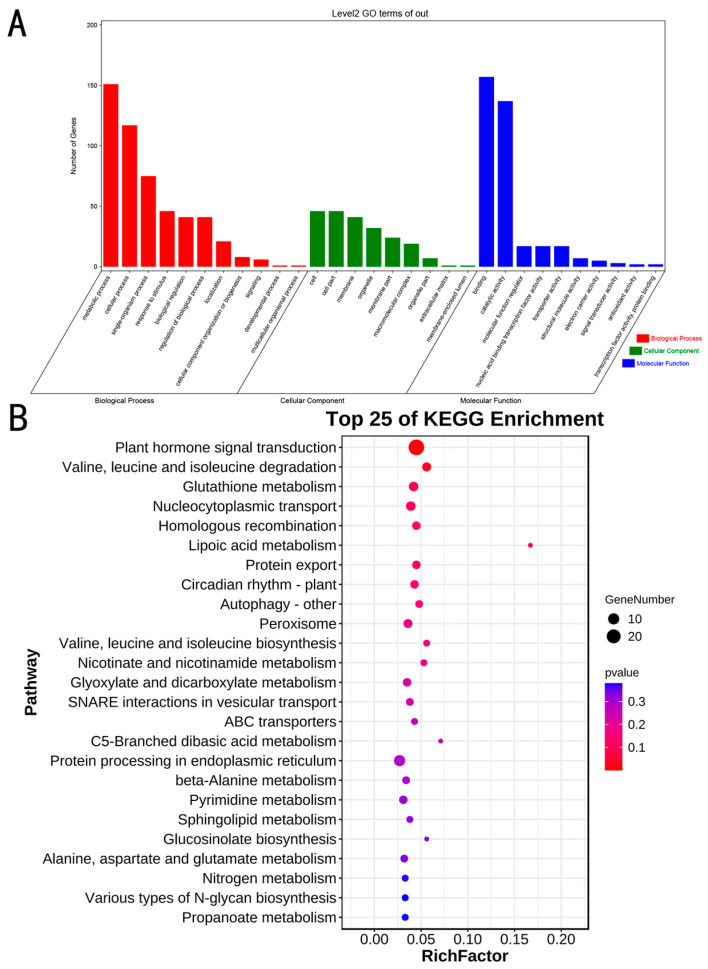
(**A**): GO annotation analysis of 797 coding genes. (**B**): KEGG enrichment analysis scatter diagram. *p* value: 0.005–0.02, *p* < 0.001 extremely significant; *p* < 0.05 significant; Gene Number: The size of the sphere represents the number of enriched genes: the larger the sphere, the more enriched genes there are.

A total of 797 coding genes were identified within a 200 kb range upstream and downstream of the 19 association sites. GO annotation analysis revealed that (1) genes enriched in biological processes were predominantly involved in metabolic and cellular processes; and (2) genes enriched in molecular functions were primarily involved in binding-related functions, followed by catalytic activity (Figure 4A). Additionally, KEGG enrichment analysis highlighted pathways related to plant hormone signal transduction, the degradation of valine, leucine, and isoleucine, and the glutathione metabolic pathway, suggesting a significant role of hormones in the soybean regeneration process (Figure 4B). Finally, after comprehensive consideration of gene function annotation, GO/KEGG functional enrichment classification, SNP significance level, and differential gene expression level, seven candidate genes were identified as the main targets for further research (Table 4).

### 2.6. Expression Analysis of Candidate Genes Related to Soybean Regeneration

**Figure 5:**
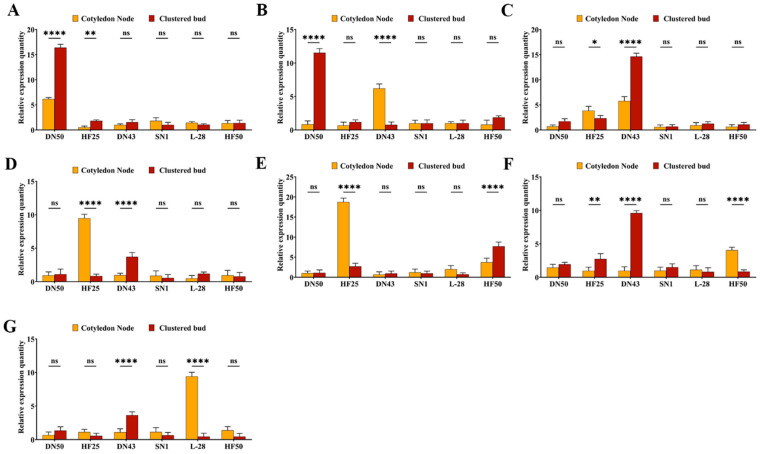
Analysis of gene expression in cotyledonary node and clustered bud. (**A**): *Glyma.12G164100*, (**B**): *Glyma.12G164700*, (**C**): *Glyma.02G006200*, (**D**): *Glyma.19G128800*, (**E**): *Glyma.04G211100*, (**F**): *Glyma.04G051300*, (**G**): *Glyma.08G319800*; “*”: *p* < 0.05, “**”: *p* < 0.01, “****”: *p* < 0.0001, “ns”: non-significant, *n* = 3.

We conducted an expression-level analysis of seven candidate genes in three soybean germplasms with high regeneration rates and three with low regeneration rates. The results indicated that the expression levels of *Glyma.12G164100* (Figure 5A), *Glyma.12G164700* (Figure 5B), *Glyma.02G006200* (Figure 5C), and *Glyma.19G128800* (Figure 5D) were highest in the cotyledon node and clustered bud of high-regeneration-rate varieties, whereas their expression levels were lower in low-regeneration-rate varieties. This suggests that these four genes play a positive regulatory role in the regeneration of the cotyledon node and clustered bud.

For *Glyma.04G211100* (Figure 5E), the highest expression level was observed in the cotyledon node of high-regeneration-rate varieties, while in the clustered bud, this gene exhibited the highest expression level in low-regeneration-rate varieties. This indicates a positive regulatory role in cotyledon node regeneration and a negative regulatory role in clustered bud regeneration. Conversely, *Glyma.04G051300* (Figure 5F) had the highest expression level in the cotyledon node of low-regeneration-rate varieties and in the clustered bud of high-regeneration-rate varieties. This gene thus plays a positive regulatory role in clustered bud regeneration and a negative regulatory role in cotyledon node regeneration.

*Glyma.08G319800* (Figure 5G) exhibited the highest expression level in low-regeneration-rate varieties for both cotyledon node and clustered bud, indicating a negative regulatory role in the regeneration of both tissues.

In conclusion, the genes *Glyma.12G164100* (*GmARF1*), *Glyma.12G164700* (*GmPPR*), *Glyma.02G006200* (*GmERF1*), and *Glyma.19G128800* (*GmAECC1*) play a positive regulatory role in the regeneration of cluster buds and cotyledon nodes in HF25, L-28, DN43, DN50, HF50, and SN1. These findings suggest that these four genes may be involved in soybean regeneration of soybean regeneration and may be crucial for subsequent analysis.

### 2.7. Subcellular Localization Results of Four Candidate Genes

**Figure 6:**
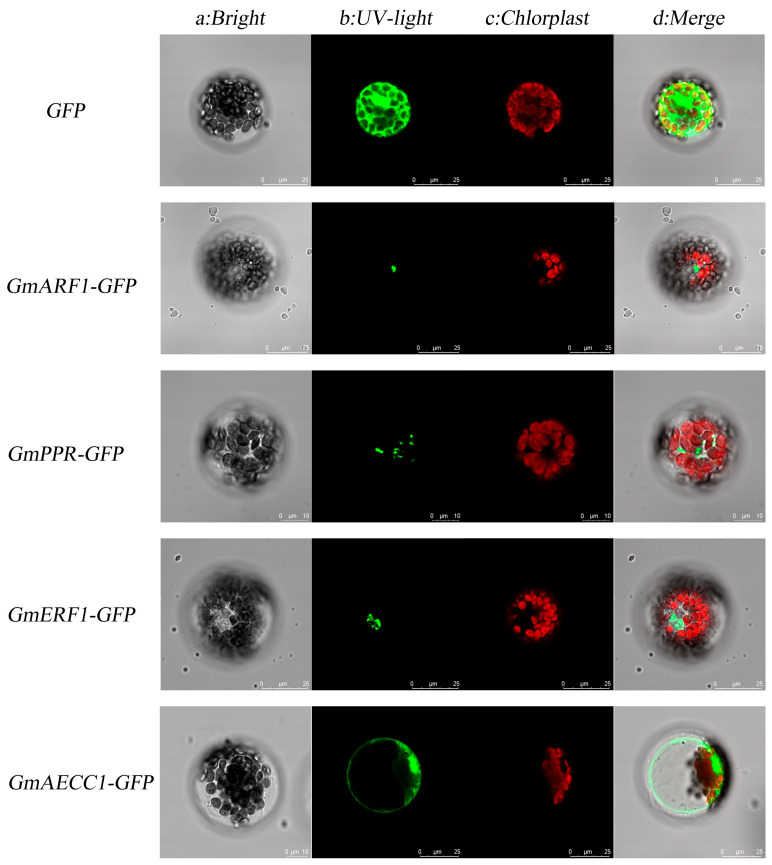
Subcellular localization of four genes; *GmARF1*-*GFP*: *GmARF1*-Green Fluorescent Protein, *GmPPR-GFP*: *GmPPR*-Green Fluorescent Protein, *GmERF1*-*GFP: GmERF1*-Green Fluorescent Protein, *GmAECC1*-*GFP*: *GmAECC1*-Green Fluorescent Protein.

The subcellular localization of the four candidate genes revealed that pCAMBIA1302-GFP was expressed in the cell membrane, nucleus, and cytoplasm. pCAMBIA1302-*GmARF1*-GFP exhibited the brightest fluorescence in the nucleus, indicating predominant nuclear localization of the encoded protein. The green fluorescence of pCAMBIA1302-*GmPPR*-GFP was observed in the cytoplasm, whereas pCAMBIA1302-*GmERF1*-GFP showed green fluorescence in the nucleus. The green fluorescence of pCAMBIA1302-*GmAECC1*-GFP was present in the cell membrane, consistent with the predicted results (Figure 6).

### 2.8. Analysis of Collinearity Between Candidate Gene Species

**Figure 7:**
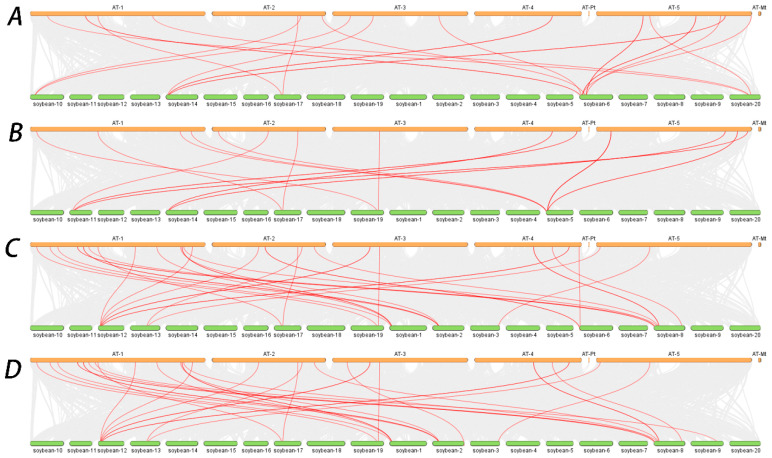
Collinearity analysis of candidate genes in soybean and *Arabidopsis thaliana*. (**A**): Collinearity of candidate genes associated with induction rate traits; (**B**): collinearity of candidate genes associated with elongation traits; (**C**): collinearity of candidate genes associated with rooting rate traits; (**D**): collinearity of candidate genes associated with seedling establishment traits. AT: *Arabidopsis thaliana*, soybean: *Glycine max*.

Seventeen pairs of homologous genes related to induction rate were identified between soybean and Arabidopsis thaliana (Figure 7A). Fourteen pairs of homologous genes were found between Arabidopsis thaliana and the candidate genes associated with bud elongation (Figure 7B). There were 26 pairs of homologous genes between the candidate genes related to rooting rate and those in Arabidopsis thaliana (Figure 7C). Similarly, 26 pairs of homologous genes were identified between the candidate genes associated with seedling rate and those in Arabidopsis thaliana, with 24 being detected (Figure 7D). The homologous gene pairs may reflect shared genomic ancestry and potential functional similarities.

### 2.9. Analysis of Regeneration Ability of Four Candidate Genes in Soybean

**Figure 8:**
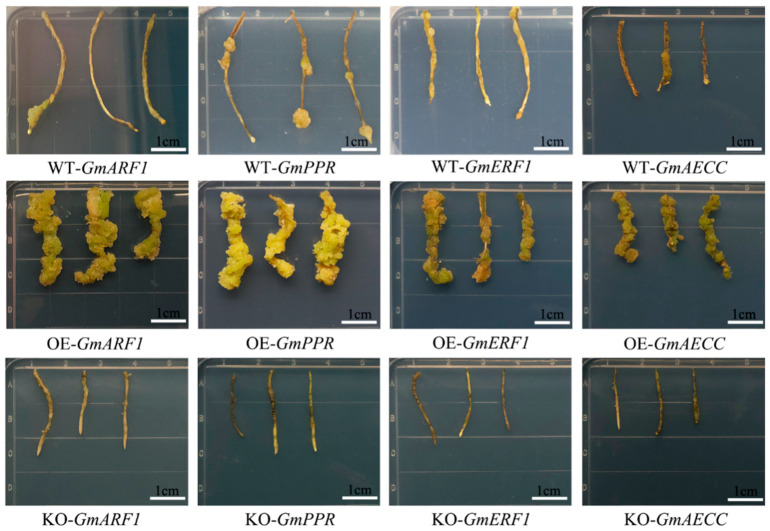
Callus formation of hairy roots. WT-*GmARF1*, WT-*GmPPR*, WT-*GmERF1*, and WT-*GmAECC*: Control hairy root culture; OE-*GmARF1*: Overexpression of *GmARF1* in hairy root culture; OE-*GmPPR*: Overexpression of *GmPPR* in hairy root culture; OE-*GmERF1*: Overexpression of *GmERF1* in hairy root culture; OE-*GmAECC*: Overexpression of *GmAECC* in hairy root culture; KO-*GmARF1*: CRISPR/Cas9-*GmARF1* hairy root culture; KO-*GmPPR*: CRISPR/Cas9-*GmPPR* hairy root culture; KO-*GmERF1*: CRISPR/Cas9-*GmERF1* hairy root culture; KO-*GmAECC*: CRISPR/Cas9-*GmAECC* hairy root culture.

The analysis of transgenic soybean hairy root callus tissue demonstrated that overexpression of the candidate genes in callus tissue enhanced callus formation compared to both the control and gene-editing groups (Figure 8). Statistical analysis of callus formation rate and density revealed that overexpression of *GmARF1*, *GmPPR*, *GmERF1*, and *GmAECC1* genes promoted hairy root regeneration. Among these, hairy roots overexpressing the *GmARF1* gene exhibited the highest regeneration potential (Table 5). The results of the one-way ANOVA for the rate of callus formation and callus density showed that the F-statistics were 2129.078 and 730.406, respectively, with a *p*-value of 0.0001 for both (which was less than 0.05). This indicates that there were significant differences in both indicators among the overexpression group, gene-edited group, and control group (Table 6).

### 2.10. Analysis of Hormone Content in Hairy Roots of Four Candidate Genes in Soybean

**Figure 9:**
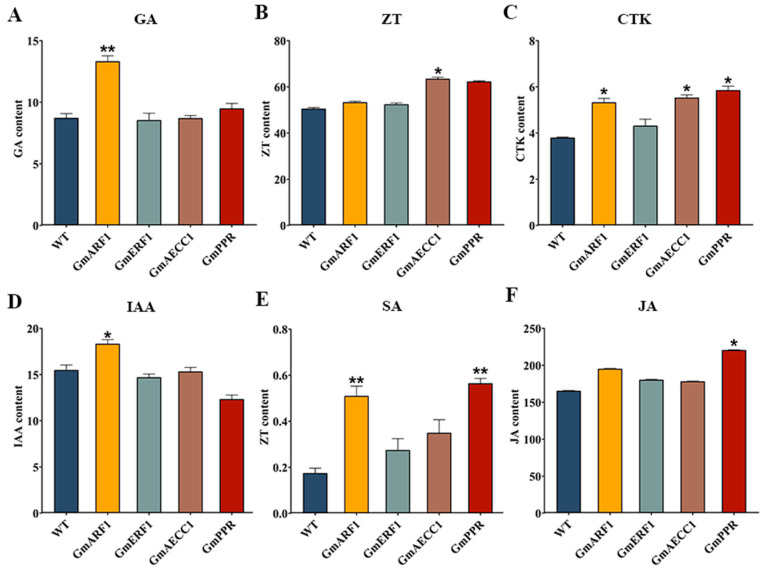
Analysis of hormone content in overexpressed soybean hairy roots. (**A**): Analysis of hormone GA content in overexpressed soybean hairy roots. (**B**): Analysis of hormone ZT content in overexpressed soybean hairy roots. (**C**): Analysis of hormone CTK content in overexpressed soybean hairy roots. (**D**): Analysis of hormone IAA content in overexpressed soybean hairy roots. (**E**): Analysis of hormone SA content in overexpressed soybean hairy roots. (**F**): Analysis of hormone JA content in overexpressed soybean hairy roots. “*”: *p* < 0.05 level difference is significant, “**”: *p* < 0.01 level difference is extremely significant, *n* = 3.

This study quantified the levels of six hormones (GA, ZT, CTK, IAA, SA, JA) in soybean hairy roots transformed with *GmARF1*, *GmERF1*, *GmAECC1*, and *GmPPR* genes. The results indicated that overexpression of these genes significantly altered hormone homeostasis, with gene-specific response patterns. The GA content in *GmARF1* overexpression lines was significantly higher than that in the wild-type (WT), indicating a potential association between *GmARF1* and GA metabolism or signaling processes (Figure 9A). However, whether this regulation is achieved by activating GA biosynthetic pathways or inhibiting its catabolism requires further verification. Additionally, there was a significant difference in IAA content between *GmARF1* overexpression lines and WT, with consistent variation trends in GA and IAA levels (Figure 9D). It is speculated that the two hormones may be involved in the regulatory network of cell elongation and differentiation, but the specific synergistic mechanism remains unclear. The responses of ZT and CTK were relatively stable. Among the ZT-promoting hormones, only *GmAECC1* overexpression in hairy roots was slightly higher than in WT, showing a significant difference (Figure 9B). CTK content was significantly increased in *GmARF1*, *GmAECC1*, and *GmPPR* hairy roots, indicating that these genes may be involved in CTK signal transduction or metabolic regulation, affecting cell division activity in hairy roots (Figure 9C). In defense-related hormone SA, gene-specific regulation was evident. The SA content of hairy roots overexpressing *GmARF1* and *GmPPR* was significantly higher than in WT, with increases of 3 times and 2.5 times, respectively, which might activate SA-mediated disease-resistance pathways. There was no significant difference in SA content between *GmERF1* and *GmAECC1* hairy roots and WT, indicating the inhibitory effect of these genes on SA synthesis or accumulation (Figure 9E). JA was significantly upregulated only in hairy roots overexpressing *GmPPR*, suggesting that *GmPPR* might specifically regulate the JA biosynthesis pathway and participate in JA-mediated stress responses (Figure 9F).

### 2.11. Expression Analysis of Four Candidate Genes Under Different Concentrations of Gibberellin Treatment

Research has shown that the expression levels of four candidate genes in soybean hairy roots induced by different concentrations of gibberellin (GA) were generally higher than those in the wild-type (WT). Among these genes, *GmAECC1* exhibited the lowest expression levels. As the treatment duration increased, the expression level of *GmARF1* showed a general downward trend, with the most significant difference compared to WT observed at 1 mg/L and 1 h of treatment. The expression peak of *GmARF1* occurred at 4 h and 24 h under a GA concentration of 2 mg/L, which might be related to the periodic activation of the GA signal. After 12 h, the expression level began to decrease, indicating an optimal concentration for the *GmARF1* response. Under 8 mg/L GA treatment, the expression peak shifted to 4–8 h and then rapidly declined, possibly due to feedback inhibition caused by high GA concentrations.

**Figure 10:**
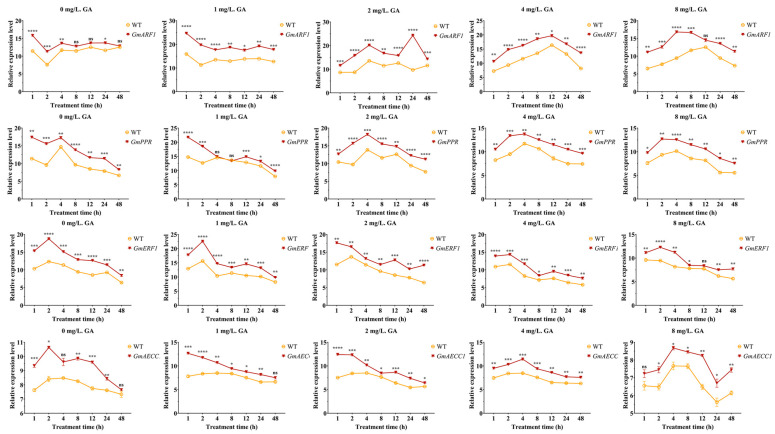
Expression levels in transgenic soybean hairy roots induced by different concentrations of gibberellin. “*”: *p* < 0.05, “**”: *p* < 0.01, “***”: *p* < 0.001, “****”: *p* < 0.0001, “ns”: non-significant, *n* = 3.

At GA concentrations of 4–8 mg/L, the expression level of *GmPPR* remained higher than that of WT, although the growth rate slowed, potentially limited by the GA-mediated growth–defense trade-off mechanism. The expression level of *GmERF1* in transgenic lines was 20–30% higher than that in WT when treated with 0 mg/L GA. It increased rapidly in the early stages of treatment (1–2 h) and gradually decreased thereafter. Treatment with 1 mg/L GA significantly enhanced its induction efficiency. When the GA concentration increased to 1 mg/L, the expression level of *GmAECC1* in transgenic lines remained significantly higher than that in WT, but the overall trend decreased compared to lower GA concentrations, indicating that the induction effect of low GA concentration on this gene was limited. As the GA concentration increased to 2–8 mg/L, the fluctuation amplitude of *GmAECC1* expression increased, reaching a significant peak at 4 mg/L, suggesting that moderate GA concentrations might more effectively activate its transcriptional response (Figure 10).

## 3. Discussion

The ability to regenerate shoots from differentiated plant tissues or to develop into whole plants is essential for plant transformation. In soybean, regeneration capacity varies significantly among different germplasms, but the genetic mechanism has not yet been verified. Researchers conducted genome-wide association studies (GWAS) on cucumber cotyledon regeneration and identified 18 significantly correlated SNP loci, further identifying three candidate genes in this region [27]. Whole genome association analysis identified 88 SNP loci related to rose bud regeneration rate and bud induction rate [28]. Previous studies on maize callus tissue regeneration through whole genome association analysis identified 130 significant SNPs [29]. Researchers identified 11 genetic loci significantly associated with callus formation in Populus tomentosa using whole genome association analysis technology and screened eight candidate genes co-expressed in other gene networks related to cell division and cell cycle [30]. In cucumber and rose, the SNP loci associated with regeneration are mainly concentrated in the cell differentiation signaling pathway. In contrast, the loci identified in this study not only cover the above-mentioned pathways but also are enriched in soybean-specific hormone response modules, a finding that provides novel insights into understanding the species-specific characteristics of legume regeneration. Different from the maize callus regeneration studies that only remain at the level of SNP locus association, this study further integrates functional verification experiments to clarify the molecular functions of candidate genes during soybean regeneration. Moreover, compared with the cell cycle-related candidate genes screened in the regeneration studies of Populus tomentosa, the genes identified in this study not only participate in the cell division process but also have cross-regulatory effects with the soybean symbiotic signaling pathway. This finding re-reveals the co-evolutionary relationship between plant regeneration traits and other biological processes, and expands the research frontier of the genetic regulatory network controlling regeneration. To the best of our knowledge, this is the first study that combines GWAS and functional verification to elucidate the genetic regulators of soybean regeneration.

Although genome-wide association analyses have been conducted on the regeneration of many species, they have not been reported in soybean regeneration. In this study, GWAS detected 66 SNP loci, including 6 associated with induction rate, 10 with elongation rate, 22 with rooting rate, and 28 with seedling rate. Notably, 19 loci appeared repeatedly across the associations. Compared to traditional selection procedures, the use of GWAS techniques can more efficiently screen genotypes with high regeneration rates and develop regeneration-related loci [29]. These results may offer a useful reference for future research on soybean regeneration. This study adopted a suggestive association threshold (−log_10_P = 3.5) to screen for loci associated with soybean regeneration-related traits. Although multiple biological validations were performed to reduce the risk of false positives, strict multiple comparison corrections such as Bonferroni or FDR were not conducted. Meanwhile, the population size of 185 germplasm resources and the SNP marker density of 21,350 may have failed to capture all weak-effect loci. Functional validation mainly relied on the hairy root callus system, and its consistency with the regeneration process of intact plants as well as adaptability across different genetic backgrounds still require further verification.

Plant hormones are integral to various stages of plant growth and development, including morphogenesis, growth, and metabolism. These hormones are synthesized in plants and operate at very low concentrations, mediated by signaling molecules to perform complex physiological functions [31]. The dynamic balance between cytokinin (CTK) and auxin (IAA) is the core of hormonal regulation of cell division and developmental reprogramming: in the induction phase, CTK activates *GmCYCD3*; 1 to initiate cell division, and a low IAA/CTK ratio drives cell re-programming [32]; in the differentiation phase, gradient accumulation of IAA guides bud differentiation, ABA downregulation relieves division inhibition, and *GmRR1* integrates signals to form a “hormone-gene-cell fate” regulatory pathway that drives regeneration [33]. ELISA relies on antigen–antibody-specific binding; while the kit manufacturer has verified extremely low cross-reactivity, potential cross-reactivity with structurally similar metabolites (e.g., hormone biosynthesis intermediates) cannot be fully excluded. It primarily provides relative quantitative data rather than absolute concentrations, limiting direct comparison of hormone levels across experimental systems. Despite sample pretreatment, complex matrix components in soybean tissues (e.g., phenolics, proteins, polysaccharides) may slightly affect detection accuracy. Further validation via LC-MS/MS is needed in subsequent studies. KEGG and GO analyses identified seven candidate genes potentially involved in soybean regeneration, several of which were associated with hormone-related pathways. Analysis of the expression patterns of these candidate genes revealed significant differences in their expression in soybean cotyledon nodes and cluster buds, indicating varying degrees of regulatory influence.

*Glyma.12G164100*, *Glyma.12G164700*, *Glyma.02G006200*, and *Glyma.19G128800* exhibited positive regulatory effects on cluster buds and cotyledon nodes. *Glyma.12G164100*, an auxin response factor, plays a crucial role in auxin signal transduction, mediates other plant hormone signal transduction pathways, and regulates plant stress resistance [34]. *Glyma.12G164700* belongs to the PPR (Pentatricopeptide repeat) gene family, which is prevalent in terrestrial plants and vital for plant growth and development, including chlorophyll and mitochondrial photosynthesis, regulation of male sterile gene expression, and embryonic development [35]. *Glyma.02G006200*, an ethylene response factor, is part of the AP2/ERF superfamily widely found in plants, and it is involved in growth, development, and responses to biotic and abiotic stresses [36,37]. *Glyma.19G128800*, an auxin efflux carrier component, is a member of the membrane transporter family. Auxin, an essential plant hormone, is synthesized mainly in coleoptiles, leaf primordia, young stems, and young roots, and is distributed throughout the plant via polar transport [38]. Consequently, these four candidate genes were selected as primary research targets.

Four candidate genes related to regeneration were overexpressed, and knockout vectors were constructed and verified by hairy root callus transformation. Compared to the control group, the overexpression of *GmARF1* and *GmPPR* genes had a more significant impact on callus differentiation and plant growth and development. Hormone content analysis in the hairy roots of overexpressed and control groups revealed that the hormone levels in the hairy roots overexpressing *GmARF1* were significantly different from those of other candidate genes. Previous studies have shown that auxin response factors are crucial for plant organ growth and seed development [39,40]. The gibberellin response element p-box, located in the promoter region of ARF1, plays a key role in drought stress response [41]. Auxin response factors also play a role in leaf senescence and may be an important component of the leaf senescence signaling pathway [42]. Additionally, two PPR family genes identified in rice significantly increased the seed-setting rate in transgenic plants under low-temperature stress, indicating enhanced stress resistance [43]. The PPR family is involved in various cellular processes during seed development [44]. Overexpression of the ERF subfamily improves cold tolerance in rice [45]. Auxin efflux vector components regulate rice tillering [46].

Under gibberellin (GA) treatment, the overexpression of the *GmARF1* gene in soybean hairy roots was higher than that of other candidate genes. Gibberellin activates gene expression in signal transduction processes, regulating plant morphogenesis, growth, and development, including seed germination, stem elongation, flowering time, and adventitious root production [39]. The DELLA protein is a key regulatory element in gibberellin signal transduction. Upon perceiving the gibberellin signal, DELLA proteins are degraded, thereby releasing their repression on growth-related processes. Conversely, structural changes in the DELLA protein can lead to gibberellin insensitivity, resulting in a GA-deficient phenotype [47]. Studies on gibberellin as an indicator of soybean regeneration are rare. Exogenous gibberellin was used to stimulate transgenic soybean hairy roots, determining the optimal concentration and application time to identify key genes involved in soybean regeneration, providing a reference for efficient soybean regeneration system establishment [48,49].

This study found that *GmARF1* was most sensitive to 4 mg/L GA, while *GmERF1* preferred 1–2 mg/L. *GmPPR* showed the smallest response amplitude to GA concentration changes, reflecting specific gene function division. Most genes responded rapidly in the early stages of GA treatment (1–4 h), with subsequent expression attenuation due to feedback regulation. In summary, GA affected the expression of the four genes in transgenic soybean hairy roots through a dual regulatory mode of concentration and time, with *GmARF1* potentially mediating rapid GA signal transduction. Overexpression of *GmARF1* significantly improved regeneration efficiency.

This study focuses on the analysis of the genetic mechanisms underlying soybean regeneration traits and has certain limitations. Firstly, only a subset of candidate genes has been functionally verified, while the candidate genes corresponding to other associated loci remain unvalidated, which is insufficient for a comprehensive elucidation of the genetic regulatory network. Therefore, future research should prioritize the use of stable transgenic soybean plants to clarify gene functions while dissecting upstream expression regulatory mechanisms. Additionally, a dynamic sampling system for key regeneration stages should be established, and integrated transcriptomic and metabolomic analyses should be performed to track the dynamic correlations among hormones, genes, and metabolites, thereby providing a more refined molecular basis for understanding the regeneration mechanism.

## 4. Materials and Methods

### 4.1. Materials

#### Plant Materials

**Figure 11:**
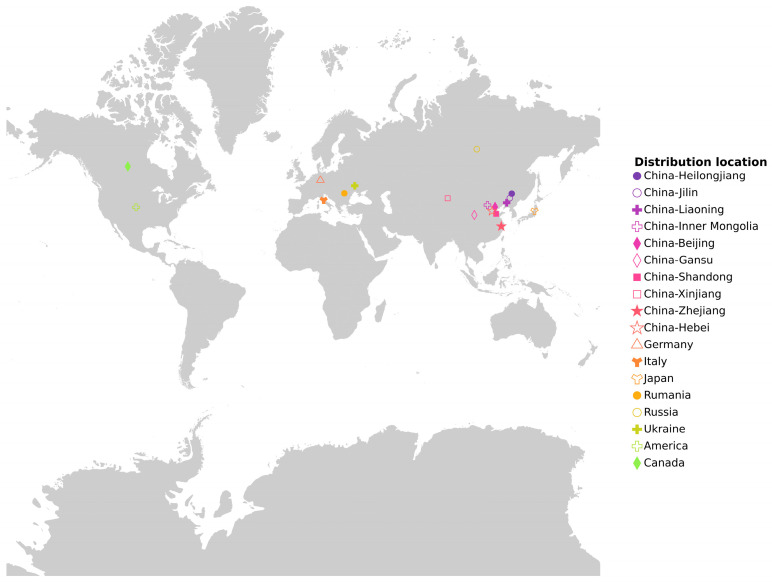
Geographical distribution of 185 soybean germplasm resources in this study.

This study utilized 185 soybean germplasm resources, including 165 domestic and 20 international varieties (Supplementary Table S3). To better understand the distribution of the research materials, a geographical distribution map of natural soybean populations was created (Figure 11). The regeneration induction rate, elongation rate, rooting rate, and seedling rate of the test materials were assessed using the soybean cotyledon node regeneration system [50].

### 4.2. Methods

#### 4.2.1. Analysis of Phenotypic Data

Phenotypic data were analyzed based on plant growth and regeneration rates were subsequently calculated. Each experimental material was treated with 40 explants, with the procedure repeated three times. The statistical indicators are as follows:

Induction rate (%) = (Budding explants/Inoculated explants) × 100%

Elongation rate (%) = (Number of explants with bud length > 2 cm/Number of inoculated explants) × 100%

Rooting rate (%) = (Number of rooting branches/Number of inoculated explants) × 100%

Seedling rate (%) = (Number of surviving transplanted seedlings/Number of inoculated explants) × 100%

Variance analysis and correlation analysis were conducted on four reproductive traits of 185 soybean germplasm resources using SPSS v27.0.1 software.

Broad-sense heritability (H^2^) was calculated as H^2^ = V_g_/V_g_ + V_e_, where (V_g_) represents genetic variance and (V_e_) denotes environmental variance.

Induction rate refers to the percentage of successfully induced callus tissues to the total number of inoculated explants within a certain cultivation period, reflecting the adaptability of induction medium, cultivation conditions, and explant genotype to the regeneration process. Elongation is an indicator of the longitudinal growth ability of explants or regenerated tissues/organs during the culture cycle, usually referring to the length growth rate of the target structure. Rooting rate refers to the percentage of regenerated seedlings (or adventitious buds) that successfully differentiate into adventitious roots under specific culture conditions and cycles, compared to the total number of inoculated regenerated seedlings or adventitious buds. Seedling formation rate refers to the percentage of materials that can successfully develop from initial explants (or intermediate regenerated tissues/shoots) into complete and viable regenerated plants during the complete regeneration culture process, compared to the total number of initial experimental materials.

#### 4.2.2. Genotype Data

Genomic DNA from all 185 soybean accessions was extracted from young leaves using the CTAB method [51]. Simplified sequencing was performed through site-specific amplified fragment sequencing (SLAF-seq) [52]. Using the digestive enzymes MseI (EC: 3.1.21.4) and HaeIII (EC: 3.1.21.4) (Thermo Fisher Scientific, Inc., Waltham, MA, USA), over 50,000 sequencing labels of 300 bp to 500 bp in length were obtained, which were evenly distributed across the unique genomic regions of the 20 soybean chromosomes. The short oligonucleotide analysis program 2 (SOAP 2) was employed to align all clean reads to the soybean reference genome, with quality control set at MAF ≥ 0.05. A genotype was defined as heterozygous when the ratio of secondary allele depth to total allele depth exceeded 1/3 [53].

#### 4.2.3. Population Structure Analysis and Linkage Disequilibrium Analysis

Based on genotype data, the genetic relationships among the 185 materials were calculated using TASSEL 5.0 software to generate a genetic relationship matrix. Cluster analysis was performed using the neighbor-joining method in MEGA X v10.2.3 software [54], and the clustering map was created and refined using the Chiplot website (https://www.chiplot.online/#Phylogenetic-Tree accessed on 25 January 2025). Genotype data were filtered and controlled using Plink with the following parameters: genotyping deletion rate (mind ≥ 0.2), minor allele frequency (MAF ≥ 0.05), and genotyping deletion rate threshold (geno ≥ 0.2). Population structure was assessed using filtered polymorphic SNP markers with ADMIXTURE (v1.3.0), specifying the K value range from 2 to 10 to determine the optimal number of ancestral populations based on the minimum cross-validation error [55]. To obtain the genetic relationship matrix, TASSEL 5.0 software was used to compute genetic relationships among the 185 materials. The neighbor-joining method in MEGA X software was employed for cluster analysis [54], and the clustering map was drawn and refined using the Chiplot website (https://www.chiplot.online/#Phylogenetic-Tree accessed on 26 January 2025).

#### 4.2.4. Screening and Prediction of Candidate Genes

Genome-wide association analysis was conducted using the mixed linear model (MLM) from the GAPIT 3.0 package in R v4.3.0 [56]. Gene annotation within the intervals was performed using the gene bioinformatics cloud platform (https://www.omicshare.com/ accessed on 1 February 2025), followed by GO enrichment analysis and KEGG pathway enrichment analysis.

#### 4.2.5. RT-qPCR Analysis of Candidate Genes

Based on gene function annotation and genome-wide association analysis, candidate genes potentially related to soybean regeneration were selected. Three soybean varieties with high and low regeneration rates were chosen from the germplasm resources as experimental materials. After 6 days of germination and 14 days of recovery, twelve samples were collected from the growth points of cotyledons and clustered buds for RT-qPCR (real-time quantitative PCR) analysis to determine the relative expression levels of the genes [57]. RT-qPCR primers were designed using Primer v5.0, and Actin was utilized as the internal reference gene (Supplementary Table S4). The RT-qPCR reaction system was configured according to the Vazyme fluorescent quantitative SYBR Green kit instructions(Nanjing Vazyme Biotech Co., Ltd., Nanjing, China). All RT-qPCR experiments were independently repeated three times. Data processing was conducted using SPSS v27.0.1, and results were analyzed using the 2^−∆∆Ct^ method. The calculation process is as follows: ∆∆Ct = ∆Ct target gene − ∆Ct internal reference gene, followed by calculation of 2^−∆∆Ct^ based on ∆∆Ct.

#### 4.2.6. Subcellular Localization Analysis

Based on the CDS sequences of candidate genes, target fragments were amplified using primers modified with 15 bp homologous sequences flanking the Nco I restriction site in the pCAMBIA1302 vector. Gene subcellular localization primers were designed using Primer v5.0 (Supplementary Table S5). The cDNA of soybean variety HF25 served as the template for amplifying the target fragments, which were subsequently recovered from the gel. The pCAMBIA1302 vector plasmid was extracted and digested with Nco I endonuclease. The target gene was then recombined into the plasmid. Arabidopsis protoplast transformation followed the Coolaber Arabidopsis protoplast preparation and transformation kit protocol.

#### 4.2.7. Collinearity Analysis Among Species

Whole genome sequences and gene structure annotation files of soybean and Arabidopsis were obtained from the Ensembl Plants database (http://plants.ensembl.org/index.html accessed on 17 March 2025). Collinearity analysis was performed using McScanX software (1.0.0) within the Biolux system, and gene collinearity analysis graphs were generated using TBtools-II software.

#### 4.2.8. Construction of Overexpression and Gene-Editing Vector

DNA extraction from soybean HF25 was conducted using the CTAB method [28]. RNA was extracted using the Trizol method [58], and DNA integrity was verified with a reverse transcription kit (TOYOBO, Osaka City, Japan) and 2% agarose gel electrophoresis. Using cDNA/DNA of HF25 as the template, candidate genes related to regeneration traits were cloned. Cloning primer information is provided in Supplementary Table S6, the amplification system in Supplementary Table S7, and the amplification procedure in Supplementary Table S8. Amplification products were detected via 2% agarose gel electrophoresis, and the target gene positions were identified (Supplementary Figure S2). The overexpression and gene-editing vectors pCRISPR/Cas9 and pCAMBIA3300 were treated with single-enzyme digestion (Supplementary Tables S9 and S10) and incubated at 37 °C for 3 h. Digestion effects were confirmed by 2% agarose gel electrophoresis. Homologous recombinases facilitated the recombination of overexpression and gene-editing plasmids (Supplementary Table S11), which were then transformed into Agrobacterium competent cells.

The basic structure of the gene-editing vector is shown in Supplementary Figure S1. This study constructed a dual-target gene-editing system using gene-editing target primers (Supplementary Table S12). Target sequences 3d and 3b were used for gene editing. The DNA dimer fragment was formed by cooling to room temperature from 90 °C for 30 s (Supplementary Table S13). The first round of the PCR reaction system and procedure are detailed in Supplementary Tables S14 and S15. Detection and gel purification were performed using 2% gel electrophoresis. In the first round of PCR, the sizes of the 3d and 3b targets were approximately 250–500 bp and 140 bp, respectively (Supplementary Figure S3). In the second round of PCR, the size of the 3d/3b PCR product was approximately 500 bp (Supplementary Figure S4). Homologous recombination of linearized Cas9 and 3d/3b fragments was performed and transformed into E. coli. The PCR reaction system (Supplementary Table S16) yielded a target band size of approximately 1000 bp (Supplementary Figure S5).

#### 4.2.9. Determination of Formation Rate and Density of Transgenic Callus Tissue

Callus formation rate (%) = (number of explants forming callus/total number of inoculated explants) × 100%

Callus density (g·cm^−3^) = fresh weight of 50 callus tissues (g)/volume of 50 callus tissues (cm^3^)

#### 4.2.10. Analysis of Hormone Content in Callus Tissue

After inducing rooting in soybean positive hairy roots for approximately 15 days, a 4 cm segment of the roots was excised, weighed, and a specific volume of PBS (pH 7.4) was added. The samples were immediately frozen in liquid nitrogen and stored until use. After thawing at 2–8 °C, PBS (pH 7.4) was added, and tissues were homogenized. The homogenates were then centrifuged for approximately 20 min at 2000–3000 rpm, and the supernatants were carefully collected. Hormone analysis was conducted according to the instructions of the enzyme-linked immunosorbent assay (ELISA) kit (Merck, Shanghai, China).

#### 4.2.11. Expression Analysis of Transgenic Hairy Roots Treated with Different Concentrations of GA

Transgenic soybean hairy roots induced for about 15 days were subjected to DNA level detection and BAR test strip analysis. GA solutions with concentrations of 0 mg/L, 1 mg/L, 2 mg/L, 4 mg/L, and 8 mg/L were prepared, using wild-type (WT) soybean as a control. Hairy roots (20 g) were immersed in GA at each concentration, and samples of wild-type and transgenic positive roots were taken at 1 h, 2 h, 4 h, 8 h, 12 h, 24 h, and 48 h. Three replicates were collected at each time point and stored at −80 °C for RNA extraction. RT-qPCR was performed following reverse transcription.

## 5. Conclusions

In this study, we utilized the cotyledon node method to identify four regeneration indices in 185 soybean germplasm resources. Genome-wide association analysis was employed to screen for four candidate genes (*Glyma.12G164100*, *Glyma.12G164700*, *Glyma.02G006200*, and *Glyma.19G128800*) associated with regeneration. Subsequent bioinformatics analysis, subcellular localization, expression analysis, gene cloning, and vector construction for overexpression and knockout experiments revealed the involvement of these candidate genes in plant growth and development. Overexpression and knockout vectors for the four candidate genes were verified using hairy root callus transformation. Notably, the expression of the *Glyma.12G164100* (*GmARF1*) gene was significantly higher than that of the other candidate genes, suggesting that *GmARF1* plays a crucial role in soybean regeneration. This finding provides a basis for further studies on soybean regeneration genes.

## Figures and Tables

**Table 1:** Analysis of variance for four regeneration indicators.

Trait	SS	Df	MS	F	*p*-Value
Ir	42.24	184	11.22	1271.92	3.67 × 10^−4^
Er	12.43	184	4.94	209.35	2.05 × 10^−5^
Rr	7.08	184	3.35	159.72	1.73 × 10^−5^
Sr	3.97	184	1.73	121.00	1.58 × 10^−4^

Note: Ir: Induction rate, Er: Elongation rate, Rr: Rooting rate, Sr: Seedling rate, SS: Sum of squares, Df: Degree of freedom, MS: Mean square, F: F value.

**Table 2:** Correlation analysis of four regeneration indicators.

Trait	Ir	Er	Rr	Sr
Ir	1	0.503 *	0.464 *	0.412 *
Er	0.503 *	1	0.930 **	0.878 **
Rr	0.464 *	0.930 **	1	0.937 **
Sr	0.412 *	0.878 **	0.937 **	1

Note: * and ** indicate significant levels at 0.05 and 0.01.

**Table 3:** Association analysis of SNPs for four regeneration traits in natural population.

Peak SNP	Chr	Position	LOD	R^2^(%)	−log_10_(p)	Trait
rs649057	2	649,057	4.66	0.25	4.66	Rr
			3.87	0.14	3.87	El
			5.72	0.24	5.72	Sr
rs48331514	4	48,331,514	3.67	1.14	3.67	Rr
			3.95	1.56	3.95	Sr
rs37438936	5	37,438,936	4.80	7.76	4.80	Rr
			3.87	5.89	3.87	El
			4.98	8.26	4.98	Sr
rs43860379	8	43,860,379	4.20	3.26	4.20	Rr
			3.76	2.57	3.76	El
			4.34	3.32	4.34	Sr
rs43890529	8	43,890,529	3.76	6.46	3.76	Rr
			3.88	6.86	3.88	Sr
rs43890567	8	43,890,567	3.61	5.75	3.61	Rr
			3.66	6.21	3.66	Sr
rs43895712	8	43,895,712	3.90	7.63	3.90	Rr
			3.74	6.49	3.74	El
			3.95	8.42	3.95	Sr
rs44290852	9	44,290,852	4.19	0.94	4.19	Rr
			3.59	0.91	3.59	El
			5.29	1.01	5.29	Sr
rs40835778	9	40,835,778	3.98	11.65	3.98	Ir
			4.66	12.97	4.66	Rr
			5.03	14.06	5.03	El
			3.95	11.07	3.95	Sr
rs12208970	10	12,208,970	4.66	0.17	4.66	Rr
			4.13	0.21	4.13	El
			5.30	0.25	5.30	Sr
rs20795004	10	20,795,004	3.92	0.41	3.92	Rr
			4.30	0.28	4.30	Sr
rs9361284	11	9,361,284	3.66	0.33	3.66	Rr
			3.71	1.39	3.71	Sr
rs15049524	14	15,049,524	4.64	12.92	4.64	Rr
			4.55	12.86	4.55	Sr
rs866769	17	866,769	5.29	2.92	5.29	Rr
			4.92	2.48	4.92	El
			5.57	3.29	5.57	Sr
rs38720070	18	38,720,070	4.62	0.78	4.62	Rr
			3.72	1.08	3.71	El
			4.81	0.66	4.81	Sr
rs46093366	18	46,093,366	3.75	0.38	3.75	Rr
			4.91	0.53	4.91	Sr
rs22268726	18	22,268,726	3.69	1.25	3.69	Rr
			3.79	0.91	3.79	Sr
rs38908816	19	38,908,816	4.05	0.48	4.05	Rr
			4.67	0.29	4.67	Sr
rs37390617	20	37,390,617	3.57	0.59	3.57	Rr
			3.91	0.87	3.91	Sr

**Table 4:** Information of the candidate genes.

Gene	Marker	Chr	Site	Trait	Functional Annotation
*Glyma.04G051300*	rs4048649	Chr04	4,162,013	Ir	cytokinin-responsive gata factor 1
*Glyma.12G164100*	rs32746557	Chr12	31,863,013	Ir	auxin response factor 1
*Glyma.12G164700*	rs32746557	Chr12	31,902,863	Ir	glutamine-rich protein 23
*Glyma.02G006200*	rs649057	Chr02	669,867	El/Sr/Rr	ethylene response factor 1
*Glyma.08G319800*	rs43860379	Chr08	43,889,780	El/Sr/Rr	cell differentiation, Rcd1-like protein
*Glyma.19G128800*	rs38757015	Chr19	38,804,185	El/Sr/Rr	auxin efflux carrier family protein
*Glyma.04G211100*	rs48331514	Chr04	48,307,226	Sr/Rr	gibberellin 20 oxidase 2

**Table 5:** Statistics of callus formation rate and density.

Treatment	Rate of Callus Formation (%)	Callus Density (g·cm^−3^)
WT	58.12 ± 2.24 ^e^	0.2534 ± 0.0005 ^e^
OE-*GmARF1*	82.50 ± 4.35 ^a^	0.5743 ± 0.0035 ^a^
OE-*GmPPR*	78.94 ± 3.44 ^b^	0.5413 ± 0.0156 ^b^
OE-*GmERF1*	75.32 ± 4.31 ^c^	0.4642 ± 0.0023 ^c^
OE-*GmAECC1*	69.84 ± 6.35 ^d^	0.3933 ± 0.0116 ^d^
KO-*GmARF1*	10.56 ± 1.25 ^f^	0.0113 ± 0.0015 ^f^
KO-*GmPPR*	9.46 ± 1.03 ^f^	0.0100 ± 0.0132 ^f^
KO-*GmERF1*	7.78 ± 1.23 ^f^	0.0091 ± 0.0006 ^f^
KO-*GmAECC1*	3.13 ± 1.24 ^g^	0.0085 ± 0.0011 ^f^

Note: Data are presented as mean ± standard error (*n* = 3). Different lowercase letters (a–g) indicate significant differences (*p* < 0.05, Duncan’s multiple range test).

**Table 6:** Single factor analysis of variance for callus formation rate and density.

		SS	DF	MS	Fisher’s	*p*
Rate of callus formation	Within-group	28,728.567	8	3591.071	2129.078	0.0001
	Between-group	30.360	18	1.687		
	Total	28,758.927	26			
Callus density	Within-group	1.418	8	0.177	730.406	0.0001
	Between-group	0.004	18	0.000		
	Total	1.422	26			

Note: SS (Sum of Squares); DF (Degrees of Freedom); MS (Mean Square); Fisher’s (F-statistic); *p* (*p* value).

## Data Availability

Data will be made available on request. Data are contained within the article and Supplementary Materials.

## Supplementary Materials

The following supporting information can be downloaded at: https://www.mdpi.com/article/10.3390/plants15010110/s1, Figure S1: The basic structure of gene editing vector Cas 9; Figure S2: Cloning of Four Candidate Genes in Soybeans. A: Gene cloning of *GmARF1*; B: Gene cloning of *GmPPR*; C: Gene cloning of *GmERF1*; D: Gene cloning of *GmAECC1*; Figure S3: The positions of 3D and 3b targets; Figure S4: The results of two rounds of PCR amplification for four genes. A: The second round of verification for *GmARF1*. B: The second round of verification for *GmPPR*. C: The second round of verification for *GmERF1*. D: The second round of verification for *GmAECC1*; Figure S5: PCR amplification results of 4 candidate gene targets. A: PCR amplification results of *GmARF1* gene target. B: PCR amplification results of *GmPPR* gene target. C: PCR amplification results of *Gm ERF1* gene target. D: PCR amplification results of *Gm AECC* gene target. Table S1: Screening of 11 varieties with high regeneration rate; Table S2: Association analysis loci for four regeneration traits in 173 soybean materials; Table S3: Source of 185 soybean accessions; Table S4: PCR primers used for expression analysis by qRT-PCR; Table S5: Subcellular localization primer; Table S6: Target gene cloning prime; Table S7: The PCR amplification system; Table S8: The PCR amplification program; Table S9: The enzyme digestion composition of *HindIII*; Table S10: The enzyme digestion composition of *Bas I*; Table S11: Ligation system of target fragment and linearized vector; Table S12: Construction of primers for CRIPRCas 9 vector; Table S13: Target Connection System; Table S14: First round PCR reaction system; Table S15: The first round of PCR validation amplification program; Table S16: Ligation system of target fragment and linearized vector.
