# Supplementary figures and images for: Genome-Wide Association Analysis of Soybean Regeneration-Related Traits and Functional Exploration of Candidate Genes
Source: Plants (Basel). 2025 Dec 31;15(1):110. doi: 10.3390/plants15010110 (PMC12787991; doi:10.3390/plants15010110)

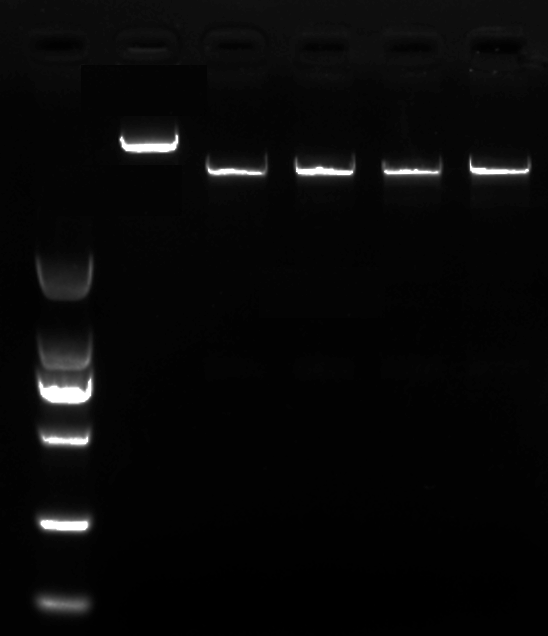

Supplement: Supplementary file 1 [file plants-15-00110-s001.zip › Figure S1 The basic structure of gene editing vector Cas 9.jpg]

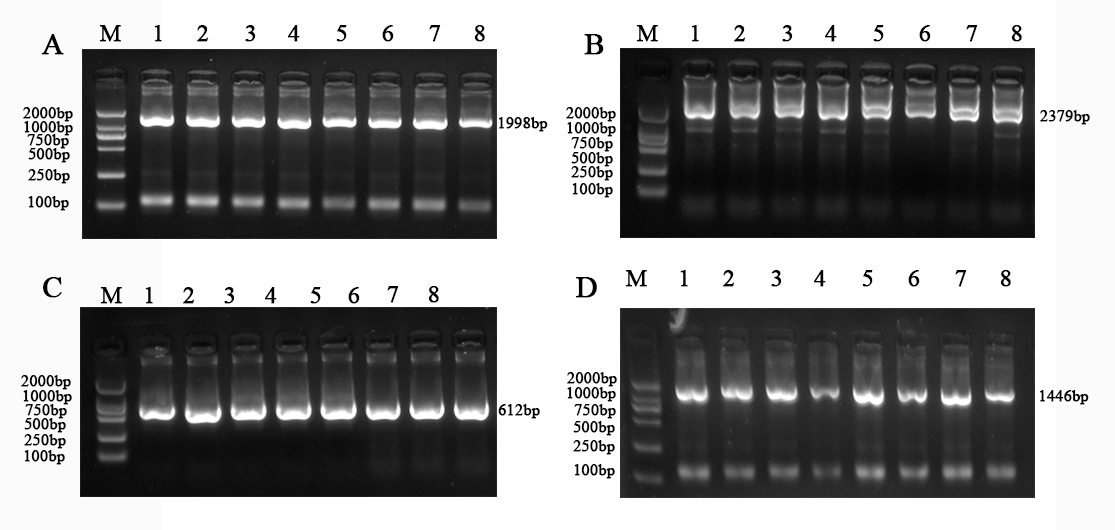

Supplement: Supplementary file 1 [file plants-15-00110-s001.zip › Figure S2 Cloning of Four Candidate Genes in Soybeans.jpg]

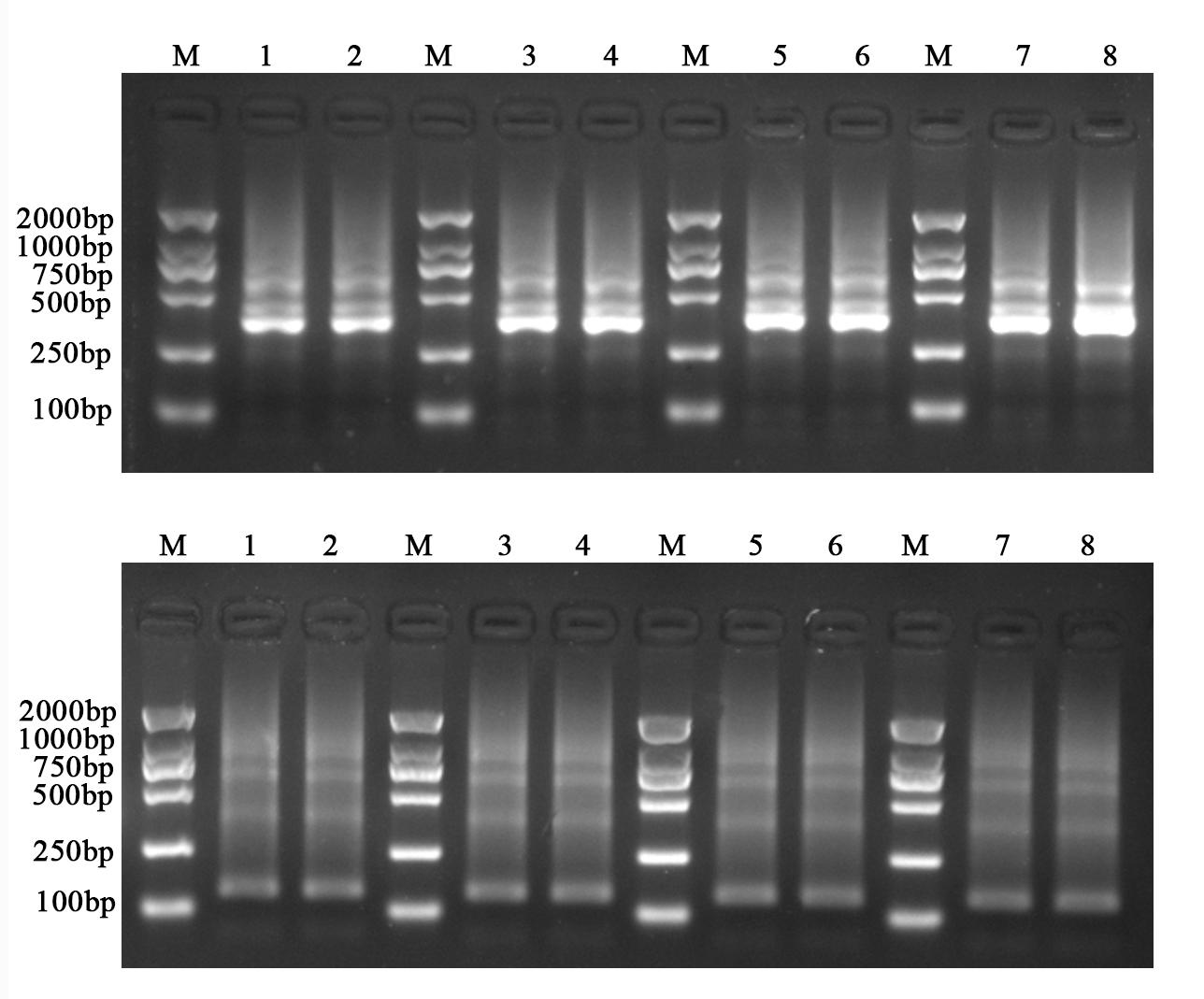

Supplement: Supplementary file 1 [file plants-15-00110-s001.zip › Figure S3 The positions of 3D and 3b targets..jpg]

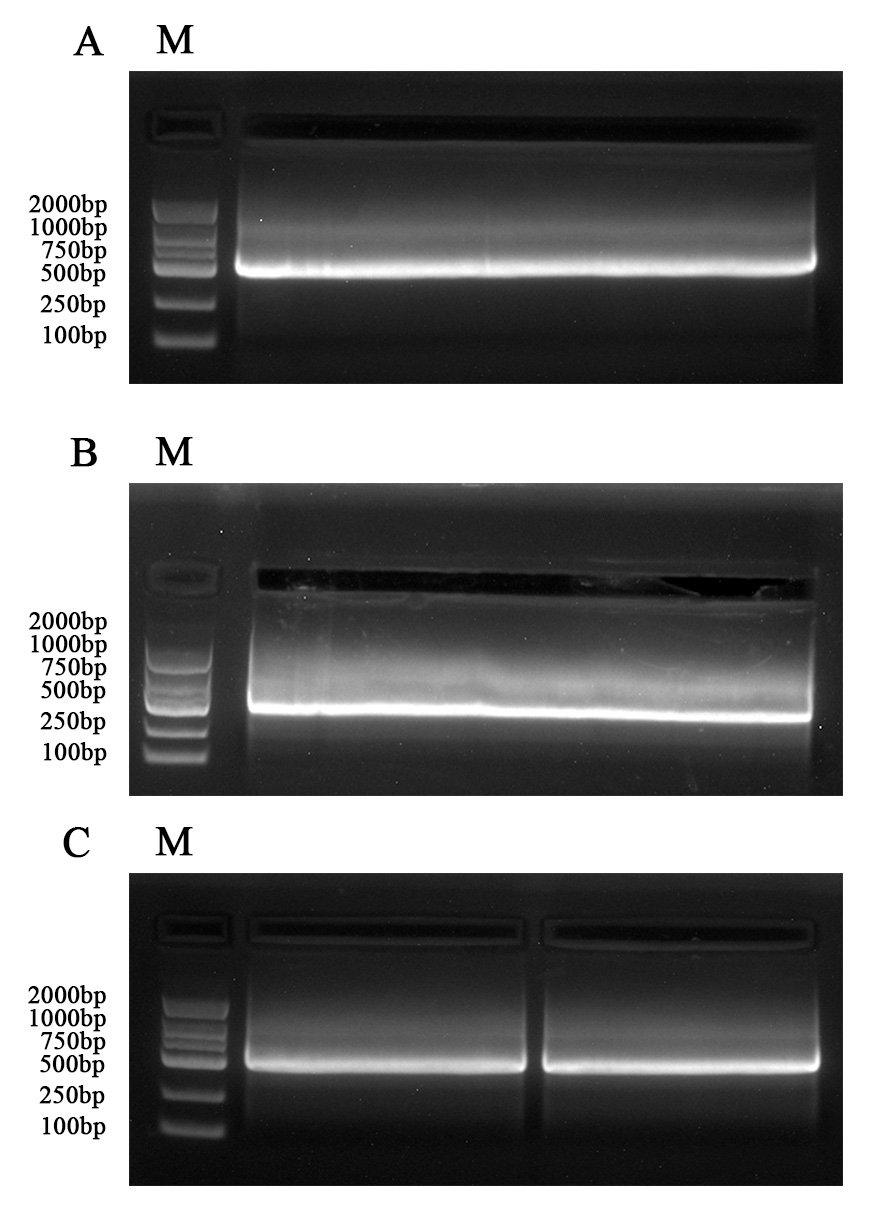

Supplement: Supplementary file 1 [file plants-15-00110-s001.zip › Figure S4 The results of two rounds of PCR amplification for four genes.jpg]

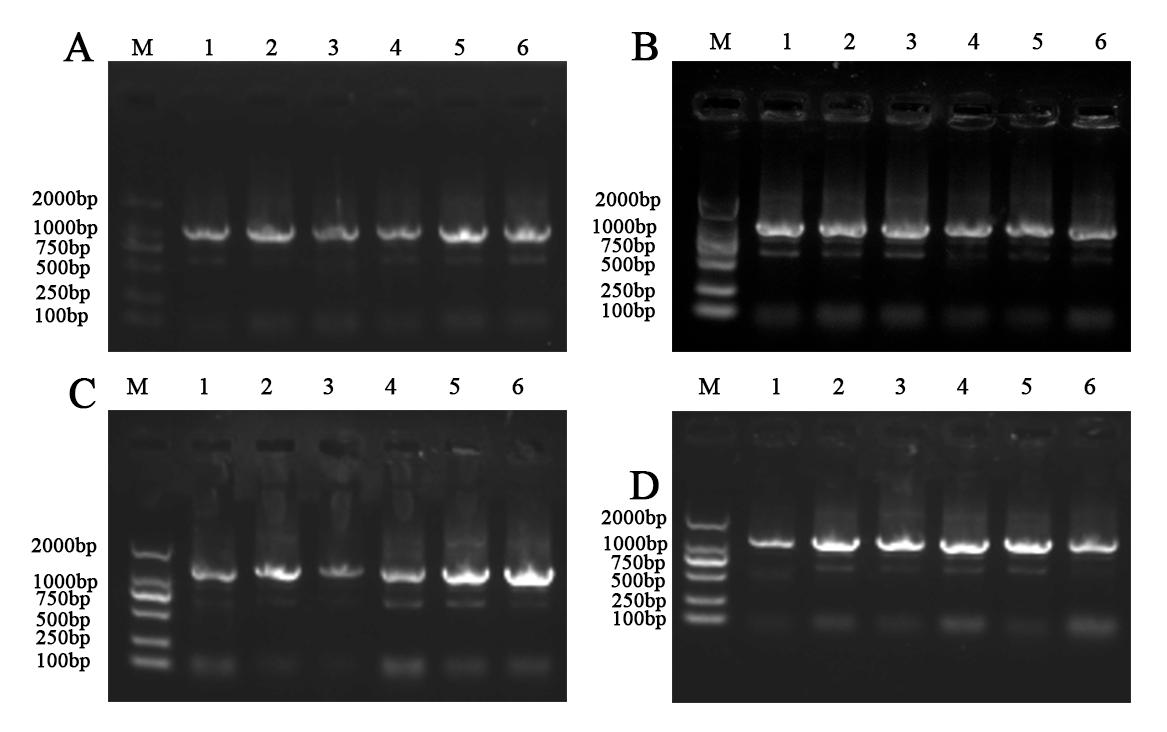

Supplement: Supplementary file 1 [file plants-15-00110-s001.zip › Figure S5 PCR amplification results of 4 candidate gene targets.jpg]
